# Altered glycolipid metabolism during acute kidney injury exacerbates renal inflammation

**DOI:** 10.1038/s41598-025-28897-4

**Published:** 2025-12-02

**Authors:** Akinori Osada, Miyako Tanaka, Yuki Sugiura, Xunmei Yuan, Shinji Yamashita, Kozue Ochi, Hiro Kohda, Ayaka Ito, Shiori Go, Tetsuya Okajima, Kenji Kadomatsu, Motoko Yanagita, Kazuhiro Furuhashi, Shoichi Maruyama, Takayoshi Suganami

**Affiliations:** 1 Department of Molecular Medicine and Metabolism, Research Institute of Environmental Medicine, Nagoya University, Nagoya, Japan; 2 Department of Nephrology, Nagoya University Graduate School of Medicine, Nagoya, Japan; 3 Department of Immunometabolism, Nagoya University Graduate School of Medicine, Nagoya, Japan; 4 Institutes of Innovation for Future Society, Institute of Nano-Life-Systems, Nagoya University, Nagoya, Japan; 5 Center for Cancer Immunotherapy and Immunobiology, Graduate School of Medicine, Kyoto University, Kyoto, Japan; 6 Department of Life and Food Sciences, Obihiro University of Agriculture and Veterinary Medicine, Obihiro, Japan; 7 Institute for Advanced Research, Nagoya University, Nagoya, Japan; 8 Institute for Glyco-Core Research, Nagoya University, Nagoya, Japan; 9 Department of Molecular and Cellular Biology, Nagoya University Graduate School of Medicine, Nagoya, Japan; 10 Department of Nephrology, Graduate School of Medicine, Kyoto University, Kyoto, Japan; 11 Institute for the Advanced Study of Human Biology (ASHBi), Kyoto University, Kyoto, Japan; 12 Center for One Medicine Innovative Translational Research (COMIT), Nagoya University, Nagoya, Japan; 13 Quantum-Based Frontier Research Hub for Industry Development, Nagoya University, Nagoya, Japan; 14 Innovative Research Center for Preventive Medical Engineering, Nagoya University, Nagoya, Japan

**Keywords:** Nephrology, Endocrinology, Inflammation

## Abstract

Acute kidney injury (AKI) can progress to chronic kidney disease (CKD), via a mechanism that is still largely unknown. We previously reported that glucosylceramide (GlcCer) acts as a damage-associated molecular pattern (DAMP) during AKI. Here, we demonstrate that renal GlcCer levels increase persistently during AKI, primarily due to oxidative stress-mediated downregulation of β-1,4-galactosyltransferase 5 (B4galt5) in proximal tubules. Using mass spectrometry, we showed that GlcCer specifically accumulated in damaged proximal tubules. Among the enzymes involved in GlcCer metabolism, B4galt5 was predominantly expressed in proximal tubules and its expression was consistently downregulated across multiple AKI models. Knockdown of B4galt5 alone was sufficient to increase GlcCer levels in cultured proximal tubular cells. Moreover, in vivo administration of GlcCer combined with free cholesterol triggered inflammatory responses via the innate immune receptor macrophage-inducible C-type lectin (Mincle). These inflammatory responses were almost abolished in Mincle-deficient mice, suggesting a specific GlcCer-Mincle pathway. Our findings indicate that B4galt5 plays a critical role in GlcCer accumulation in necrotic tubules following AKI. Specifically, we propose that dying proximal tubules alter their glycolipid metabolism to generate DAMPs, highlighting B4galt5 as a potential therapeutic target for preventing the AKI-to-CKD transition.

**Supplementary Information:**

The online version contains supplementary material available at 10.1038/s41598-025-28897-4.

## Introduction

Acute kidney injury (AKI) is characterized by a rapid decline in renal function. It is associated with high mortality rates, prolonged hospital stays, and elevated medical costs^1,2^. Initially, renal function was thought to return to almost normal levels after recovery from AKI. However, accumulating evidence has indicated a high risk of transition from AKI to chronic kidney disease (CKD)^3,4^, and its possible underlying mechanisms have included tubular damage and cell death, inflammation, hypoxia, and interstitial fibrosis^5–7^. Among them, metabolic abnormalities in the proximal tubules have attracted substantial attention. The proximal tubules consume large amounts of energy to actively reabsorb and excrete water, electrolytes, and nutrients. AKI impairs ATP dynamics in the proximal tubules, which significantly affects renal prognosis^8^. Because proximal tubules are also actively involved in glucose metabolism, sodium-glucose cotransporter-2 (SGLT2) inhibitors have been proposed to improve the prognosis of AKI as well as CKD^9–11^. Furthermore, abundant expression of peroxisome proliferator-activated receptors (PPARs) suggests a crucial role of lipid metabolism in the proximal tubules^12,13^. However, how glucose and lipid metabolism is regulated in the proximal tubules during the AKI-to-CKD transition remains unclear.

We previously elucidated the pathogenic mechanisms underlying cell death–triggered chronic inflammation in obesity and metabolic syndrome^14–18^. In principle, dead cells are rapidly eliminated by phagocytic cells such as macrophages, and when this process is delayed or impaired, it causes inflammation. During the reparative phase of AKI, we observed a unique histological structure, in which necrotic tubules were surrounded by macrophages expressing the innate immune sensor macrophage-inducible C-type lectin (Mincle), thereby inducing sustained inflammation. We also identified β-glucosylceramide (GlcCer) as an endogenous Mincle ligand, which accumulated in the necrotic tubules. Combined with free cholesterol, GlcCer exerts its proinflammatory effects through Mincle in macrophages in vitro^19^. However, whether it can induce renal inflammation in vivo remains to be elucidated.

GlcCer is the starting point of various glycolipid molecules, including sphingolipids. GlcCer is synthesized in the Golgi apparatus, undergoes glycosylation, and is transported to the plasma membrane^20^. Thus, GlcCer is thought to be localized primarily within the cell and acts as a danger signal (damage-associated molecular pattern: DAMP) when exposed extracellularly during proximal tubular necrosis following AKI. GlcCer has been reported to be implicated in polycystic kidney disease, diabetic nephropathy, and lupus nephritis, whereby it may regulate cell death, inflammation, and endoplasmic reticulum stress^21–24^. However, the mechanism by which renal injury alters GlcCer metabolism remains unclear. While standard mass spectrometric analysis cannot distinguish between GlcCer and galactosylceramide (GalCer), which make up hexosylceramide (HexCer)^25^^,^ GalCer shows no Mincle ligand activity^19^. Accordingly, the specific changes of GlcCer must be clarified in the kidney.

In this study, we provide the first evidence that the amount of GlcCer changes dynamically during AKI in mice. The underlying mechanism includes the role of β-1,4-galactosyltransferase 5 (B4galt5), an enzyme that metabolizes GlcCer into lactosylceramide^20^. Moreover, we demonstrate the in vivo proinflammatory action of GlcCer through Mincle. This study reveals the molecular basis for alterations in lipid metabolism occurring in the proximal tubules following AKI, which will lead to new therapeutic targets for preventing the AKI-to-CKD transition.

## Results

### Glycolipid levels in the kidneys after ischemia–reperfusion injury

**Fig. 1:**
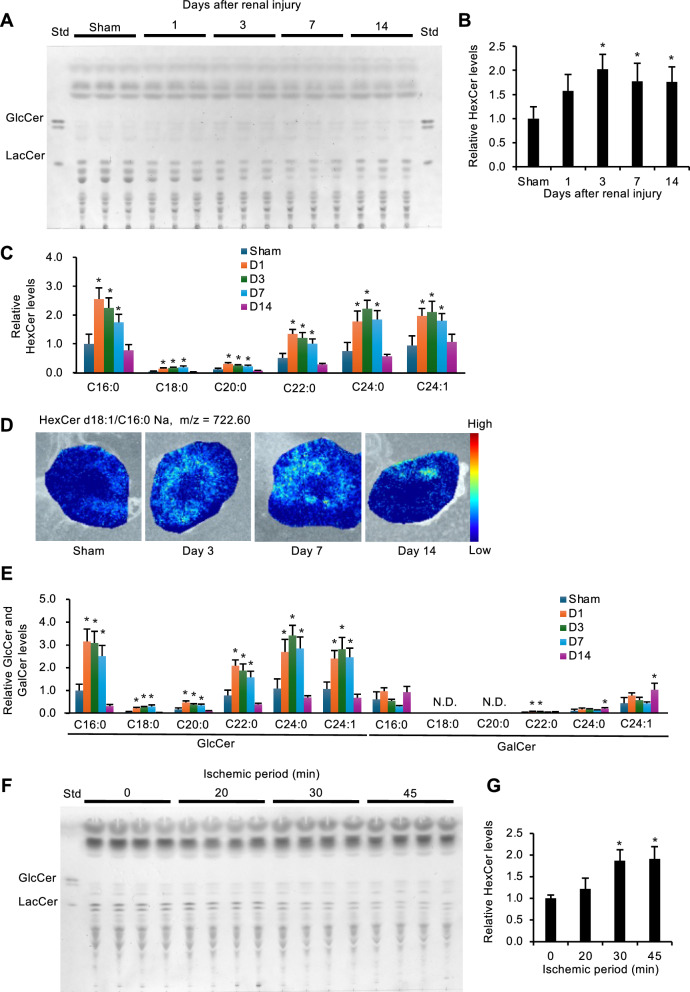
Glycolipid levels in the kidneys after ischemia–reperfusion injury. (**A**–**E**) Male C57BL/6 J mice were subjected to renal ischemia–reperfusion injury (ischemia for 30 min) and analyzed on the indicated days. (A, B) Thin-layer chromatography (TLC) analysis of extracted renal lipids. Representative TLC chromatogram (**A**) and hexosylceramide (HexCer) quantification (**B**). HexCer includes glucosylceramide (GlcCer) and galactosylceramide (GalCer). The lane labeled “Std” corresponds to the reference standard. *n* = 5–6. (**C**) Measurement of HexCer in extracted renal lipids using LC–MS/MS. Various types of HexCer containing different fatty acids were measured on the indicated days. *n* = 4–5. (**D**) MS imaging of HexCer (d18:1/C16:0) in the kidneys on the indicated days. (E) Measurement of GlcCer and GalCer in extracted renal lipids using LC–MS/MS in HILIC mode. *n* = 4–5. GlcCer and GalCer with different fatty acids were separately measured on the indicated days. (**F**, **G**) Male C57BL/6 J mice were subjected to renal ischemia–reperfusion injury (ischemia for 0–45 min) and analyzed on day 3. Representative TLC chromatograms (**F**) and the quantitative data (**G**) for HexCer levels. *n* = 4. Data are expressed as mean ± SD. Statistical differences were measured by one-way ANOVA followed by Tukey–Kramer post hoc test. *P < 0.05 vs. Sham or 0 min.

To investigate changes in renal levels of GlcCer, an endogenous Mincle ligand^19^, following AKI, we employed a mouse model of renal ischemia–reperfusion injury (ischemia for 30 min). In this study, kidney samples were collected on day 1 (acute phase), 3, 7 (reparative phase), and 14 (chronic phase). Thin-layer chromatography (TLC) revealed that the renal HexCer levels, primarily consisting of GlcCer, gradually increased, peaked on day 3, and remained persistently elevated (Fig. 1A, B). Liquid chromatography-tandem mass spectrometry (LC–MS/MS) analysis showed that various types of HexCer containing different fatty acids increased similarly during the experimental period (Fig. 1C). MS imaging revealed that these HexCer changes were localized exclusively to the proximal tubules of the corticomedullary region, where tissue damages occurred following renal injury (Fig. 1D). GalCer, a type of HexCer, is believed to be less abundant than GlcCer in the kidneys^26^. Since GlcCer and GalCer share the same m/z value, it is technically challenging to distinguish them using LC–MS/MS analysis. Thus, we specifically measured the GlcCer and GalCer levels using LC–MS/MS in hydrophilic interaction chromatography (HILIC) mode^27,28^. The GlcCer levels were markedly increased during AKI, whereas the GalCer levels did not change throughout the experimental period (Fig. 1E). On the other hand, ceramide levels slightly increased on day 1, and lactosylceramide levels significantly increased from day 1 to day 7, although both remained much lower than GlcCer levels (Fig. S1). Accumulation of HexCer was evident during the severe renal damage with longer ischemic periods (Fig. 1F, G). Collectively, these findings indicate an increase in GlcCer levels in the injured region of the kidneys after ischemia–reperfusion injury.

### Altered expression of GlcCer metabolism–related enzymes in AKI models

**Fig. 2:**
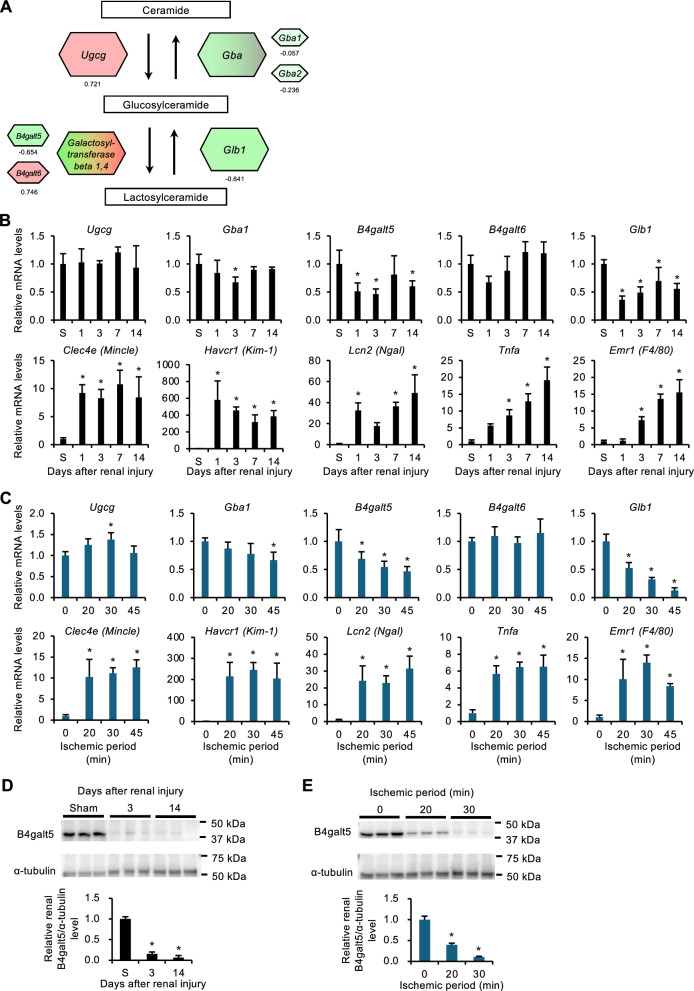
Expression of GlcCer metabolism–related enzymes in the kidneys after ischemia–reperfusion injury. (**A**) Schematic illustration of the GlcCer metabolism pathway. Each number represents mRNA expression level of GlcCer metabolism–related genes in the kidneys 3 days after ischemia–reperfusion injury (ischemia for 30 min), as determined by transcriptional analysis. Values represent Log2 fold changes; red and green indicate up and down, respectively. (**B**) Time course of mRNA expression levels of GlcCer metabolism–related genes in the kidneys. S indicates the sham group. *n* = 5–6. (**C**) Effect of ischemic period on mRNA expression levels of GlcCer metabolism–related genes in the kidneys. *n* = 4. (**D**) Time course of protein levels of GlcCer metabolism–related enzymes in the kidneys. S indicates the sham group. *n* = 3. (E) Effect of ischemic period on protein levels of GlcCer metabolism–related enzymes in the kidneys. *n* = 3. Data are expressed as mean ± SD. Statistical differences were measured by one-way ANOVA followed by Tukey–Kramer post hoc test. *P < 0.05 vs. S or 0 min.

**Fig. 3:**
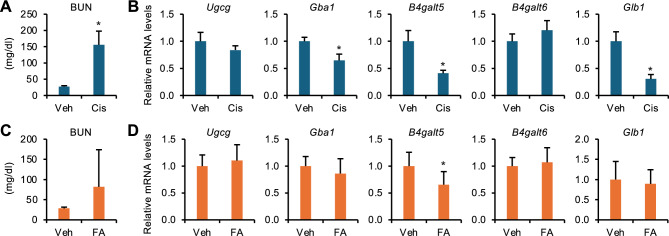
Expression of GlcCer metabolism–related genes in various AKI models. Male C57BL/6 J mice were subjected to cisplatin (Cis)- and folic acid (FA)–induced AKI. The kidneys were analyzed 3 days after the injection of cisplatin or vehicle (Veh; saline) (A, B), or folic acid or vehicle (Veh; 300 mM sodium bicarbonate buffer) (**C**, **D**). (**A**, **C**) Serum BUN concentrations. (**B**, **D**) Expression levels of GlcCer metabolism–related genes of the kidney. *n* = 5–6 (**A**, **B**) or 5–10 (**C**, **D**). Data are expressed as mean ± SD. Statistical differences were measured by unpaired *t*-test. *P < 0.05 vs. Veh.

Next, we examined the expression profiles of GlcCer metabolism–related genes in the AKI models. Transcriptomic analysis on the kidney 3 days after ischemia–reperfusion injury revealed downregulation of *B4galt5*, a major enzyme involved in GlcCer metabolism that transfers galactose to GlcCer to form lactosylceramide (Fig. 2A). These changes were validated by real-time PCR throughout the experimental period (Fig. 2B). *B4galt5* mRNA levels markedly decreased on day 1 and remained low until day 14. B4galt5 protein levels also markedly decreased on day 3 and day 14 (Fig. 2D and Fig. S3A). mRNA levels of *Clec4e* (*Mincle*) and proinflammatory cytokines such as tumor necrosis factor-α (*Tnfa*) significantly increased on day 1 (Fig. 2B), consistent with our previous report^19^. The extent of the decrease in *B4galt5* mRNA and protein levels was dependent on the ischemic period and inflammatory changes (Fig. 2C, E) and Fig. S3B). Using recent single-nucleus RNA sequencing analysis with a renal ischemia–reperfusion injury model^29^, we showed that *B4galt5* was expressed predominantly in the proximal tubules of the renal parenchyma, whereby it markedly decreased after renal injury (Fig. S2). On the other hand, UDP-glucose ceramide glucosyltransferase (*Ugcg*), glucocerebrosidase (*Gba1*), and *B4galt6* showed only marginal changes in expression (Fig. 2B, C). β-galactosidase (*Glb1*) mRNA levels significantly decreased after renal injury (Fig. 2B, C). Given that Glb1 counteracts B4galt5 in the regulation of GlcCer metabolism^30,31^, downregulation of *Glb1* expression may not contribute to renal GlcCer accumulation following ischemia–reperfusion injury. We further validated the expression profiles of GlcCer metabolism–related genes in other AKI models (Fig. 3). In the cisplatin-induced model, expression levels of *B4galt5*, *Gba1*, and *Glb1* were all significantly downregulated, whereas only *B4galt5* expression was reduced in the folic acid–induced model. These findings led us to hypothesize that B4galt5 plays a key role in the regulation of GlcCer metabolism in AKI.

### Oxidative stress regulates gene expression involved in GlcCer metabolism

**Fig. 4:**
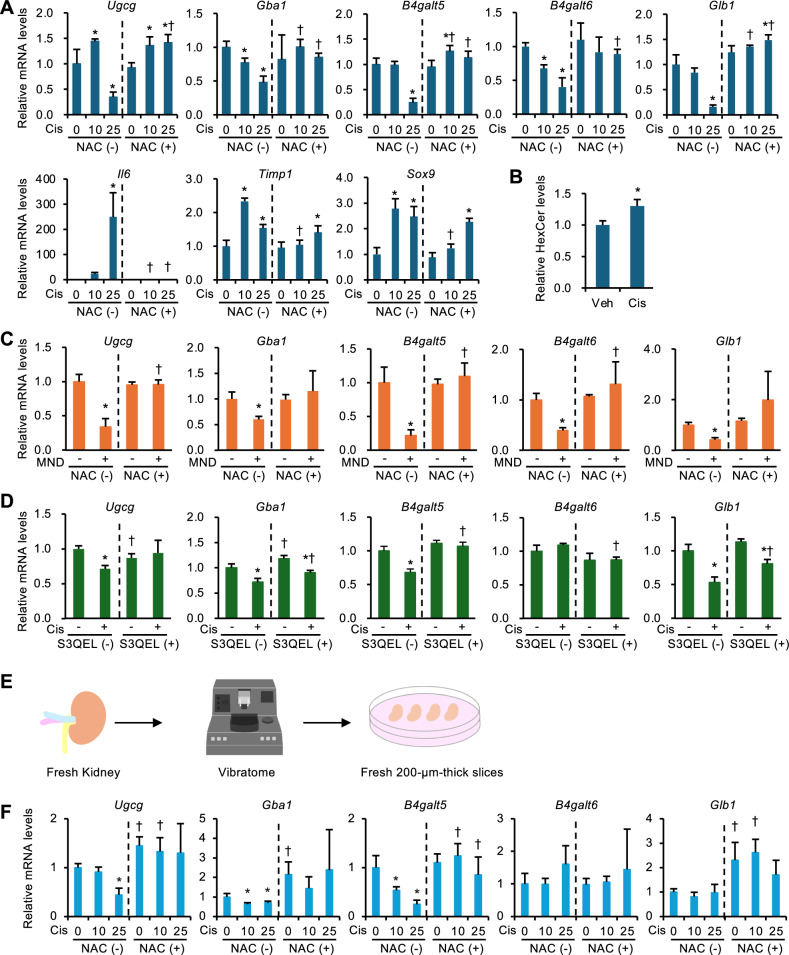
Oxidative stress regulates *B4galt5* expression in vitro. (**A**) Expression of GlcCer metabolism–related genes in cultured mouse renal tubular epithelial cell line (mProx24 cells) treated with cisplatin (Cis; 10 or 25 µM) in the presence or absence of N-acetylcysteine (NAC; 5 mM) for 24 h. *n* = 4. (**B**) HexCer quantification. mProx24 cells were treated with cisplatin (Cis; 25 µM) or vehicle (Veh; PBS) for 24 h. *n* = 4. TLC image used for quantification is shown in Fig. S6. (**C**) Expression levels of GlcCer metabolism–related genes in mProx24 cells treated with menadione (MND; 10 µM) in the presence or absence of NAC (5 mM) for 9 h. *n* = 4. (**D**) Expression levels of GlcCer metabolism–related genes in mProx24 cells treated with cisplatin (Cis; 25 µM) in the presence or absence of S3QEL (10 µM) for 24 h. *n* = 3–4. (**E**) Schematic illustration of the renal thin tissue slices experiment. (**F**) Expression levels of GlcCer metabolism–related genes in renal thin tissue slices treated with cisplatin (Cis; 10 or 25 µM) in the presence or absence of NAC (5 mM) for 24 h. *n* = 4. Data are expressed as mean ± SD. Statistical differences were measured by one-way ANOVA followed by Tukey–Kramer post hoc test. *P < 0.05 vs. 0 µM. Differences between the samples treated with the same cisplatin or menadione concentration in the presence or absence of NAC or S3QEL were evaluated by unpaired *t*-test. †*P* < 0.05.

There is substantial evidence that oxidative stress plays a major pathogenic role in the AKI-to-CKD transition^6,32^. To investigate the mechanisms regulating GlcCer metabolism–related genes, we used mProx24 cells, a mouse proximal tubular epithelial cell line, and treated them with cisplatin to obtain an in vitro model of AKI. Cisplatin treatment significantly suppressed the mRNA levels of *B4galt5* and *Glb1*, while increasing those of renal inflammation- and injury-related genes, such as *Il-6*, *Timp1*, and *Sox9* (Fig. 4A). These changes were almost completely inhibited by co-treatment with N-acetylcysteine, suggesting the involvement of oxidative stress. TLC analysis confirmed that cisplatin treatment significantly increased HexCer levels (Fig. 4B). To further investigate the role of oxidative stress in the regulation of *B4galt5* expression, we examined the effect of menadione, a synthetic form of vitamin K3 that is commonly used as an oxidative stress inducer, on *B4galt5* expression in mProx24 cells. Treatment with menadione markedly suppressed *B4galt5* mRNA levels, and this suppression was abolished by co-treatment with N-acetylcysteine (Fig. 4C and Fig. S4A). Similar results were observed for mRNA levels of other GlcCer metabolism–related enzymes. Moreover, we used S3QEL, a selective inhibitor of superoxide production from mitochondrial complex III of the electron transport chain, to investigate the involvement of mitochondrial oxidative stress in the regulation of *B4galt5* expression. Treatment with S3QEL almost completely inhibited the downregulation of *B4galt5* expression (Fig. 4D), whereas it did not affect the upregulation of *Il-6* (Fig. S4B). These findings provide additional insights into the molecular mechanisms underlying *B4galt5* expression. To better mimic in vivo conditions, we conducted kidney slice culture experiments (Fig. 4E). As with mProx24 cells, cisplatin treatment significantly decreased *B4galt5* mRNA levels, which was abolished by co-treatment with N-acetylcysteine (Fig. 4F). These findings indicate that oxidative stress elicited by renal injury downregulates *B4galt5*.

### B4galt5 regulates GlcCer production in vitro

**Fig. 5:**
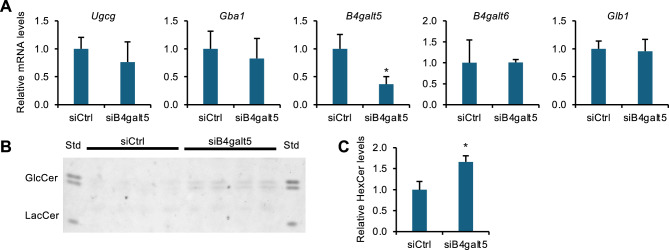
*B4galt5* regulates GlcCer production in vitro. The mProx24 cell line was treated with *B4galt5* siRNA and control siRNA for 48–64 h. (**A**) Expression levels of GlcCer metabolism–related genes. (B, **C**) TLC analysis of extracted cellular lipids. Representative TLC chromatogram (**B**) and HexCer quantification (**C**). TLC image used for quantification is shown in Fig. S6. *n* = 4. Data are expressed as mean ± SD. Statistical differences were measured by unpaired *t*-test *P < 0.05 vs. siCtrl.

To investigate the role of B4galt5 in GlcCer production, we knocked down *B4galt5* in mProx24 cells and found that it did not affect mRNA levels of other GlcCer metabolism–related genes (Fig. 5A). Under these experimental conditions, TLC revealed that *B4galt5* knockdown significantly increased HexCer levels in mProx24 cells (Fig. 5B, C). Taken together, our data suggest that downregulation of *B4galt5* expression following AKI leads to GlcCer accumulation in severely damaged and dying renal proximal tubules.

### GlcCer induces renal inflammation through Mincle

**Fig. 6:**
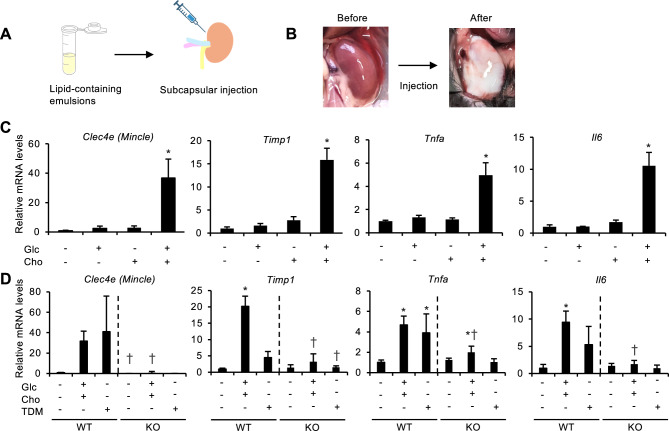
GlcCer induces renal inflammation through Mincle. (**A**) Schematic illustration of the experimental protocol. Emulsion containing GlcCer plus cholesterol, trehalose-6,6'-dimycolate (TDM), or vehicle (Veh; emulsion alone) was injected into the subcapsular space of the kidneys of wild-type (WT) and *Mincle*-deficient (KO) mice. (**B**) Representative gross appearance of the kidney after the subcapsular injection. (**C**) Expression levels of inflammation-related genes 3 days after the subcapsular injection of GlcCer and/or free cholesterol in WT mice. *n* = 5. Glc and Cho indicate GlcCer and cholesterol, respectively. (**D**) Expression levels of inflammation-related genes 3 days after the subcapsular injection of GlcCer plus free cholesterol or TDM. *n* = 3–5. Data are expressed as mean ± SD. Statistical differences were measured by one-way ANOVA followed by Tukey–Kramer post hoc test. *P < 0.05 vs. the control group without stimulation. Differences between the genotypes in the respective condition were evaluated by unpaired *t*-test. †*P* < 0.05 vs. the corresponding treatment in each experiment.

Previously, we screened lipid extracts from the kidneys following renal ischemia–reperfusion injury and identified GlcCer as an endogenous Mincle ligand^19^. The ligand activity of GlcCer per se was mild, while it markedly increased when GlcCer was combined with free cholesterol abundant in the lipid extracts. Although GlcCer and free cholesterol accumulated in necrotic tubules following renal injury, this ligand activity was only evaluated in in vitro experiments. In this study, an emulsion containing GlcCer plus free cholesterol, trehalose-6,6'-dimycolate (TDM), an exogenous Mincle ligand from *Mycobacterium tuberculosis*, or vehicle was injected into the subcapsular space of the kidneys (Fig. 6A, B). Injection of GlcCer in combination with free cholesterol significantly upregulated inflammation-related genes in wild-type mice, including *Clec4e (Mincle)*, *Timp1*, *Tnfa*, and *Il-6* on day 3, whereas GlcCer or free cholesterol alone did not show any apparent effect (Fig. 6C). Notably, GlcCer combined with free cholesterol upregulated these inflammatory genes to levels similar to those upon TDM treatment (Fig. 6D). Moreover, these effects were almost abolished in the kidneys of *Mincle*-deficient mice (Fig. 6D). These in vivo findings were essentially consistent with our previous in vitro data obtained with cultured macrophages^19^. Taken together, our data indicate that GlcCer serves as an endogenous Mincle ligand in vivo and is sufficient to induce renal inflammation.

## Discussion

Using LC–MS/MS in HILIC mode, we specifically detected GlcCer in the kidneys and demonstrated, for the first time, how its levels change dynamically during AKI. Notably, we showed that GlcCer can induce an inflammatory response through Mincle in vivo. The underlying mechanisms include sustained downregulation of B4galt5 following AKI. Because the conversion of GlcCer to lactosylceramide requires ATP^20^^,^ hypoxic conditions in the corticomedullary area, where the proximal tubules are located, may contribute to GlcCer accumulation in the acute phase of AKI. GlcCer levels remain high even after hypoxic conditions resolve during the reparative phase^8^, suggesting that altered gene expression involved in glycolipid metabolism, in addition to reduced ATP levels, contributes to the persistent increase in GlcCer. Given the consistent downregulation of *B4galt5* in several AKI models and its proximal tubule–selective expression, B4galt5 appears to play a critical role in GlcCer accumulation in necrotic tubules. Moreover, the above changes in glycolipid metabolism could explain the production of DAMPs by dying cells in disease models, although the molecular mechanisms remain largely unknown in vivo. Since systemic *B4galt5*-deficient mice are embryonically lethal due to placental dysplasia or hematopoietic failure^33–35^, the generation of proximal tubule–specific *B4galt5*-deficient mice will help elucidate the pathogenic role of GlcCer accumulation in the AKI-to-CKD transition.

Among the seven B4galt subtypes, only B4galt5 and B4galt6 catalyze lactosylceramide synthesis^36^. While both enzymes overlap in the central nervous system^37^, their expression patterns differ in the kidneys: B4galt5 is abundant in the proximal tubules,whereas B4galt6 shows only faint protein expression. Hence, B4galt5 is the predominant isoform in the kidneys. These observations are consistent with our finding that *B4galt5* knockdown alone is sufficient to increase GlcCer levels in cultured proximal tubular cells. Although the regulatory mechanisms controlling B4galt5 expression remain largely unknown, we found that oxidative stress potently downregulates *B4galt5 *in vitro. Proximal tubules exist in a physiologically hypoxic state, and oxidative stress serves as a common pathogenetic mechanism in AKI of various etiologies^38–41^. Future studies investigating the mechanism by which oxidative stress downregulates B4galt5 expression in dying proximal tubules may lead to the development of novel therapeutic strategies to maintain B4galt5 levels during renal injury.

Our findings reveal the complex and temporally dynamic role of GlcCer metabolism during AKI. Although we identified B4galt5 as a key regulator, the involvement of other GlcCer metabolism–related enzymes, such as Ugcg, Glb1, Gba, and B4galt6, requires further investigation, particularly using conditional *B4galt5*-deficient mice. We also need to understand the entire GlcCer metabolic pathway, including ceramide, during AKI. Indeed, Dupre et al*.* demonstrated that inhibition of GlcCer synthesis worsens AKI, suggesting a protective role of converting toxic ceramides to GlcCer^42^. Our findings do not contradict this protective role,rather, they reveal an additional pathological role of accumulated GlcCer in the later phases. Notably, in our previous study, *Mincle* deficiency protected against renal injury in the reparative/chronic phases (from day 3 to day 28), while showing no difference in the acute phase (day 1)^19^. Given that GlcCer accumulating in dead proximal tubules acts on Mincle expressed by infiltrating macrophages, these observations suggest a dual role of GlcCer metabolism: initially protective against ceramide toxicity, but subsequently proinflammatory through Mincle activation.

GlcCer was initially identified as a Mincle ligand in the culture supernatant of dead cells^43^. We previously identified GlcCer as an endogenous Mincle ligand, which accumulated in necrotic tubules during the reparative phase of AKI, and demonstrated that it exhibited strong Mincle-activating properties in the presence of free cholesterol in vitro^19^. In this study, we confirmed the in vivo Mincle-activating property when GlcCer was administered under the renal capsule. In AKI with tubular necrosis, excessive accumulation of GlcCer in proximal tubules may be exposed to the extracellular space and activate Mincle on the surrounding macrophages, thereby inducing sustained inflammation. Although it is still unclear how the combination of GlcCer and free cholesterol shows a drastic effect on the Mincle activity, free cholesterol per se does not directly act on murine Mincle^19^. Future studies examining how *B4galt5* deficiency affects GlcCer accumulation and subsequent renal injury in AKI models will provide definitive evidence for this mechanism.

In summary, our findings demonstrate that the persistent increase in HexCer during AKI results primarily from GlcCer accumulation, at least partly, driven by oxidative stress–mediated downregulation of *B4galt5* in renal proximal tubules (Fig. S5). We also show that GlcCer, in combination with free cholesterol, induces Mincle-mediated inflammatory responses in the kidneys in vivo. While various DAMPs mediate cell death–triggered inflammatory responses, this study is unique in highlighting how dying cells alter their glycolipid metabolism to accumulate DAMPs. Future studies using temporally regulated, proximal tubule–specific *B4galt5*-deficient mice will elucidate both the physiological function of B4galt5 in the kidneys and its pathological role in the AKI-to-CKD transition.

## Methods

### Reagents

All reagents were purchased from Sigma-Aldrich (St Louis, MO, USA) or Nacalai Tesque (Kyoto, Japan) unless otherwise noted.

### Animals

*Mincle*-deficient mice on the C57BL/6 J genetic background were generously provided by Dr. Shizuo Akira (Osaka University)^44^. C57BL/6 J mice were purchased from CLEA (Tokyo, Japan). The mice were housed in a temperature-, humidity-, and light-controlled environment with a 12-h light/dark cycle. They had ad libitum access to water and standard chow (CE-2, 343.1 kcal per 100 g, 12.6% energy derived from fat; CLEA Japan). All animal experiments were conducted in accordance with the ethical guidelines and approved by the Committee on the Ethics of Animal Experiments of Nagoya University (approval number R240021).

### Mouse models of AKI

Renal ischemia–reperfusion injury was induced using 8–12-wk-old male mice. Mice were anesthetized with medetomidine (0.75 mg/kg), butorphanol (5 mg/kg), and midazolam (4 mg/kg). The left kidneys were exposed through small flank incisions, and the kidney pedicle was clamped for 20, 30 or 45 min. After the clamp was released (reperfusion), the mice were administered atipamezole (0.75 mg/kg) and observed for up to 14 days. During surgery, mice were placed on a 37 °C heat pad. For drug-induced AKI models, cisplatin or folic acid was administered to 8-week-old male C57BL/6 J mice at a dose of 15 mg/kg (1 mg/mL in saline) or 250 mg/kg (dissolved in 300 mM sodium bicarbonate buffer), respectively. Serum blood urea nitrogen (BUN) concentrations were measured using a biochemical analyzer (DRI-CHEM NX500V; FUJIFILM Wako Pure Chemicals, Tokyo, Japan).

### In vivo Mincle stimulation

Emulsion containing TDM (50 μg; Cat# tlrl-tdm , Invivogen, San Diego, CA, USA) or Synthetic β-GlcCer (d18:1/C24:1) (100 μg; Cat# 860549P, Avanti Polar Lipids, Alabaster, AL, USA) combined with cholesterol (5 mg) was prepared as described previously^45^ and 100 µL was directly injected into the renal subcapsular space. Three days after the injection, the kidneys were collected and subjected to gene expression analysis.

### Cell and tissue culture

For cell culture experiments, mProx24 cells, a mouse proximal tubular cell line^46^, were kindly provided by Dr. Takeshi Sugaya (CIMIC Holdings, Tokyo, Japan) and maintained under 5% CO2 at 37 °C in Dulbecco’s modified Eagle’s medium (DMEM) supplemented with 10% fetal bovine serum (FBS), penicillin, and streptomycin. For tissue culture experiments, the kidneys of 8-week-old male C57BL/6 J mice were cut into 200-μm slices using a vibratome (Neo Linear Slicer MT,Dosaka, Kyoto, Japan). For a cisplatin-induced in vitro AKI model, mProx24 cells or tissue slices were treated with 10 or 25 µM cisplatin (5 mM in PBS) with or without 5 mM N-acetylcysteine (Cat# 017–05,131, FUJIFILM Wako Pure Chemicals) for 24 h. For an oxidative stress experiments, mProx24 cells were treated with 10 μM menadione (20 mM in DMSO; Cat#134–08,131, FUJIFILM Wako Pure Chemicals, Osaka, Japan) with or without 5 mM N-acetylcysteine for 9 h. Moreover, mProx24 cells were treated with 25 µM cisplatin (5 mM in PBS) with or without 10 μM S3QEL (1 mM in DMSO; Cat#18,556, Cayman Chemical) for 24 h.

### siRNA Transfection

*B4galt5* was knocked down using MISSION siRNA. mProx24 cells were transfected with 50 nM si*B4galt5* (MISSION siRNA ID: SASI_Mm01_00134140; Sigma-Aldrich) or negative control siRNA (MISSION siRNA Universal Negative Control #2, Cat# SIC002; Sigma-Aldrich) using Lipofectamine RNAiMAX (Cat# 13,778,030, Thermo Fisher Scientific, Waltham, MA, USA). After 24 h of incubation, the cells were retransfected with the same siRNA and incubated for an additional 24–40 h prior to analysis.

### Quantitative real-time PCR

Quantitative real-time PCR was performed as previously described^15^. In brief, total RNA was extracted from the tissues or cultured cells using Sepasol reagent, and 10 ng of cDNA was used for real-time PCR amplification with SYBR GREEN detection protocol in a thermal cycler (StepOne Plus,Thermo Fisher Scientific). Primers used in this study are listed in Supplementary Table 1. The data were normalized to *36B4* levels and analyzed using the comparative cycle threshold method.

### Analysis of glycosphingolipids

Total lipids were extracted from the kidneys and cultured cells using the Bligh and Dyer method^47^. Subsequently, total extracted lipids were subjected to mild alkalization treatment and analyzed by TLC. For TLC standards, synthetic β-GlcCer (d18:1/C24:1), (d18:1/C16:0) (Cat# 860539P, Avanti Polar Lipids), and LacCer (d18:1/C24:1) (Cat# 860597P, Avanti Polar Lipids) were used. Glycosphingolipids were detected and analyzed using the ChemiDoc Imaging system (Bio-Rad, Hercules, CA, USA) and Image Lab software (version 5.2.1,Bio-Rad). For Fig. 1B and Fig. S6A, two independent TLC images that were run in parallel under identical experimental conditions were analyzed and used for quantification, whereas other figures were quantified from a single image. Glycosphingolipids levels were normalized to the protein concentration determined using the Pierce BCA Protein Assay Kit (Thermo Fisher Scientific). Full images of TLC are shown in Fig. S6.

### MALDI-MS imaging

Matrix-assisted laser desorption/ionization mass spectrometry imaging (MALDI-MSI) was performed as previously described^48^, with minor modifications. Briefly, frozen kidney tissues were sectioned at a thickness of 8 μm using a cryomicrotome (CM3050, Leica Microsystems, Tokyo, Japan) and mounted onto indium tin oxide (ITO)-coated glass slides (Bruker Daltonics GmbH, Leipzig, Germany). A matrix solution of 2,5-dihydroxybenzoic acid (50 mg/mL in 80% ethanol) was manually applied using an airbrush (Procon Boy FWA Platinum, Mr. Hobby, Tokyo, Japan). To ensure consistent analyte extraction and co-crystallization, the matrix was uniformly sprayed across multiple tissue sections simultaneously. MALDI-MSI was conducted using a timsTOF fleX instrument (Bruker Daltonics) under the following acquisition parameters: positive ion mode detection, pixel resolution of 80 μm, 200 laser pulses per pixel at a frequency of 10 kHz, and a laser power setting of 50%.

### LC–MS/MS

The amount of GlcCer in kidney tissue extracts was quantified by liquid chromatography-tandem mass spectrometry (LC–MS/MS) using a Q-Exactive Focus Orbitrap mass spectrometer (Thermo Fisher Scientific) coupled with an UltiMate 3000 RSLC system (Dionex, Thermo Fisher Scientific). Hydrophilic interaction liquid chromatography (HILIC) was performed using a DCpak P4VP column (150 × 2.1 mm, 3.0 μm; DAICEL, Japan) maintained at 40 °C and operated at a flow rate of 0.2 mL/min. The mobile phases consisted of (A) methanol/water (95:5, v/v) containing 10 mM ammonium acetate and 0.2% acetic acid, and (B) acetonitrile/methanol/water (95:2:3, v/v/v) containing 10 mM ammonium acetate and 0.2% acetic acid. A 2 μL aliquot of kidney lipid extract was injected. The gradient program was as follows: 0 min, 100% B; 30 min, 0% B; 31 min, 0% B; 31.1 min, 100% B; and 40 min, 100% B. The sample tray was maintained at 4 °C throughout the analysis. The HPLC conditions were determined based on a previous report^27^.

### Western blotting

Western blotting was performed as described^15^. In brief, kidneys were lysed in RIPA (radioimmunoprecipitation assay) buffer supplemented with protease inhibitor cocktail and phosphatase inhibitor cocktail. Proteins were separated by SDS–PAGE (sodium dodecyl sulfate–polyacrylamide gel electrophoresis), and membranes were cut between approximately 100 kDa and 20 kDa to conserve antibody use. The membranes were incubated with the primary antibodies listed in Supplementary Table 2, followed by incubation with anti-rabbit IgG, HRP (horseradish peroxidase)-conjugated antibody (diluted 1:3,000,#7074; Cell Signaling Technology, Danvers, MA, USA) or anti-mouse IgG, HRP-linked antibody (diluted 1:4,000; #7076; Cell Signaling Technology) as secondary antibodies. Immunoblots were detected and analyzed with ECL Select (RPN2235; Cytiva, Marlborough, MA, USA) and the LuminoGraph imaging system (ATTO, Tokyo, Japan). Representative uncropped blots are shown in Fig. S7.

### cDNA microarray analysis

Analysis of kidney expression data from wild-type mice subjected to renal ischemia–reperfusion injury on day 3 was performed using the Gene Expression Omnibus (GEO) under accession number GSE153321 (https://www.ncbi.nlm.nih.gov/geo/query/acc.cgi?acc=GSE153321). Data were processed using the Affymetrix Microarray Analysis Suite 5.0 (MAS5) algorithm. A pathway diagram of ceramide metabolism in Fig. 2A was generated using the Path Designer in IPA (QIAGEN).

### Statistical analysis

Data are presented as means ± SD. Statistical significance was defined as P < 0.05. Statistical analyses were performed using JMP version 18.2.0 (SAS Institute Inc., Cary, NC, USA). Comparisons among multiple groups were performed using analysis of variance (ANOVA) followed by Tukey–Kramer post hoc test. Comparisons between two groups were performed using the unpaired *t*-test.

### Study approval

All methods were carried out in accordance with the ARRIVE guidelines. Animal experiments were conducted in accordance with the guidelines for the care and use of laboratory animals at Nagoya University. All protocols were approved by the Animal Care and Use Committee of the Research Institute of Environmental Medicine at Nagoya University (approval number R240021).

## Supplementary Information

Supplementary Information.

## Data Availability

The data are available from the corresponding authors upon reasonable request. The data on cDNA microarray and single-nucleus RNA-seq analyses from wild-type mice subjected to renal ischemia–reperfusion injury are publicly available in the Gene Expression Omnibus (GEO) under accession number GSE153321, and GSE139107 (https://www.ncbi.nlm.nih.gov/geo/query/acc.cgi?acc=GSE139107), respectively.
