# Supplementary material for: Altered glycolipid metabolism during acute kidney injury exacerbates renal inflammation
Source: Sci Rep. 2025 Dec 2;16:147. doi: 10.1038/s41598-025-28897-4 (PMC12765023; doi:10.1038/s41598-025-28897-4)
Supplement: Supplementary file 1 — Supplementary Information. [file 41598_2025_28897_MOESM1_ESM.pdf]

Supplementary Information

Altered glycolipid metabolism during acute kidney injury exacerbates renal inflammation

Akinori Osada, Miyako Tanaka, Yuki Sugiura, Xunmei Yuan, Shinji Yamashita, Kozue Ochi, Hiro Kohda, Ayaka Ito, Shiori Go, Tetsuya Okajima, Kenji Kadomatsu, Motoko Yanagita, Kazuhiro Furuhashi, Shoichi Maruyama, Takayoshi Suganami

Contents:

Supplementary Table 1. List of primers used in this study.

Supplementary Table 2. List of antibodies used in this study.

Supplementary Figure 1. Glycolipids levels after renal ischemia–reperfusion injury.

Supplementary Figure 2. Public snRNA data on expression levels of GlcCer metabolism–related genes during renal ischemia–reperfusion injury.

Supplementary Figure 3. Protein levels of GlcCer metabolism–related enzymes in the kidneys after ischemia–reperfusion injury.

Supplementary Figure 4. Expression of genes in cultured renal tubular epithelial cells.

Supplementary Figure 5. Schematic illustration of this study.

Supplementary Figure 6. Full images of TLC used for quantification in Figures 1A, F, 4B, and 5B, C

Supplementary Figure 7. Full images of Western blots used for quantification in Figure 2D, E and Supplementary Figure 3B, D.

Supplementary Table 1. List of primers used in this study.

Gene (mouse)	Forward primer (5-3′)	Reverse primer (5-3′)
B4galt5	GGCGGAGAAGATGACTTGTG	CGATGGTGAGGAATGGACTTG
B4galt6	ATTGCATCCGTTTCTCCTTCC	CAGGCCCTAAACTTCCATGA
Clec4e (Mincle)	ACCAAATCGCCTGCATCC	CACTTGGGAGTTTTTGAAGCATC
Emr1 (F4/80)	CTTTGGCTATGGGCTTCCAGTC	GCAAGGAGGACAGAGTTTATCGTG
Gba1	GACCAACGCTTGCTGCTAC	ACAGCAATGCCATGAACGTA
Glb1	GGATGGACAGCCATTCCGAT	CAGGGCACGTACATCTGGATA
Havcr1 (Kim-1)	TCCACACATGTACCAACATCAA	GTCACAGTGCCATTCCAGTC
Hmox1	AACTAGCCCAGTCCGGTGAT	TCTCGGCTTGGATGTGTACC
Il6	CCAGAGATACAAAGAAATGATGG	ACTCCAGAAGACCAGAGGAAAT
Lcn2 (Ngal)	ACGGACTACAACCAGTTCGC	GGGACAGCTCCTTGGTTCTT
Sox9	GTGCAAGCTGGCAAAGTTGA	TGCTCAGTTCACCGATGTCC
Timp1	CATCACGGGCCGCCTA	AAGCTGCAGGCACTGATGTG
Tnfa	ACCCTCACACTCAGATCATCTTC	TGGTGGTTTGCTACGACGT
Ugcg	AGTTTCAATCCAGAATGATCAGG	CATTCTGAAATTGGCTCACAAAT
36b4	GGCCCTGCACTCTCGCTTTC	TGCCAGGACGCGCTTGT

Supplementary Table 2. List of antibodies used in this study.

	Vendor		incubation
B4galt5	Aviva Systems Biology ARP46500_P050	rabbit polyclonal	1:1000, 4°C, overnight
B4galt6	Proteintech 20148-1-AP	rabbit polyclonal	1:2000, 4°C, overnight
Gba	Aviva Systems Biology ARP61322_P050	rabbit polyclonal	1:1000, 4°C, overnight
Glb	Proteintech 15518-1-AP	rabbit polyclonal	1:1000, 4°C, overnight
Ugcg	Bioss bs-8593R-TR	rabbit polyclonal	1:1000, 4°C, overnight
α-Tubulin	Cell Signaling Technology #3873	mouse monoclonal	1:4000, 4°C, overnight

Supplementary Figure Legends

Supplementary Figure 1. Glycolipid levels after renal ischemia–reperfusion injury. Measurement of GlcCer, GalCer, Ceramide, and LacCer in extracted renal lipids using LC-MS/MS in HILIC mode. Data are shown as intensities (arbitrary units). $n = 4–5$. Data are expressed as mean ± SD. Statistical differences were measured by one-way ANOVA followed by Tukey–Kramer post hoc test. *$P < 0.05$ vs. Sham.

Supplementary Figure 2. Public snRNA data on expression levels of GlcCer metabolism–related genes during renal ischemia–reperfusion injury. Single nucleus RNA-seq data of mouse IRI kidney. Dot plot displaying gene expression patterns of cluster-enriched markers, and bar plot displaying composition of clusters by groups. The Gene Expression Omnibus accession number GSE139107 (https://www.ncbi.nlm.nih.gov/geo/query/acc.cgi?acc=GSE139107) was used for the analysis. ATL, thin ascending limb of loop of Henle; Bil, bilateral; CNT, connecting tubule; CPC, principle cells of collecting duct in cortex; CTAL, thick ascending limb of loop of Henle in cortex; DCT, distal convoluted tubule; DTL, descending limb of loop of Henle; EC, endothelial cells; Fib, fibroblasts; ICA, type A intercalated cells of collecting duct; ICB, type B intercalated cells of collecting duct; MD, macula densa; Mø, macrophages; MPC, principle cells of collecting duct in medulla; MTAL, thick ascending limb of loop of Henle in medulla; PEC, parietal epithelial cells; Per, pericytes; Pod, podocytes; PT-S1, S1 segment of proximal tubule; PT-S2, S2 segment of proximal tubule; PT-S3, S3 segment of proximal tubule; Uro, urothelium.

Supplementary Figure 3. Protein levels of GlcCer metabolism–related enzymes in the kidneys after ischemia–reperfusion injury. (A) Time course of protein levels of GlcCer metabolism–related enzymes in the kidneys. $n = 3$. **(B)** Effect of ischemic period on protein levels of GlcCer metabolism–

related enzymes in the kidneys. $n = 3$.

Supplementary Figure 4. Expression of genes in cultured renal tubular epithelial cells. (A) Effect of menadione, an oxidative stress inducer, and N-acetylcysteine (NAC) on mRNA expression levels in the cultured renal tubular epithelial cells (mProx24 cells). mProx24 cells were treated with menadione (MND; 10 μM) in the presence or absence of NAC (5 mM) for 9 h. $n = 4$. **(B)** Effect of cisplatin and S3QEL, a selective inhibitor of superoxide production from mitochondrial complex III of the electron transport chain, on mRNA expression levels in mProx24 cells. mProx24 cells were treated with cisplatin (Cis; 25 μM) in the presence or absence of S3QEL (10 μM) for 24 h. $n = 3–4$. Data are expressed as mean ± SD. Statistical differences were measured by one-way ANOVA followed by Tukey-Kramer post hoc test. *$P < 0.05$ vs. 0 μM. Differences between the samples treated with the same cisplatin or menadione concentration in the presence or absence of NAC were evaluated by unpaired *t*-test. †$P < 0.05$.

Supplementary Figure 5. Schematic illustration of this study. Reactive oxygen species (ROS) generated during AKI suppresses B4galt5 expression in the Golgi apparatus and inhibits LacCer synthesis, resulting in GlcCer accumulation in renal tubular epithelial cells. Thus, GlcCer is released extracellularly as DAMPs upon cell death and acts as a ligand for Mincle on macrophages, thereby promoting inflammation.

Supplementary Figure 6. Full images of TLC used for quantification in Figures 1A, F, 4B, and 5B, C. (A) Full images of TLC corresponding to Figure 1A. **(B)** Full images of TLC corresponding to Figure 1F. **(C)** Full images of TLC corresponding to Figure 4B. **(D)** Full images of TLC corresponding to Figure 5B and C.

Supplementary Figure 7. Full images of Western blots used for quantification in Figure 2D, E and Supplementary Figure 3A, B. (A) Full images of Western blots corresponding to Figure 2D and Supplementary Figure 3A. **(B)** Full images of Western blots corresponding to Figure 2E and Supplementary Figure 3B.

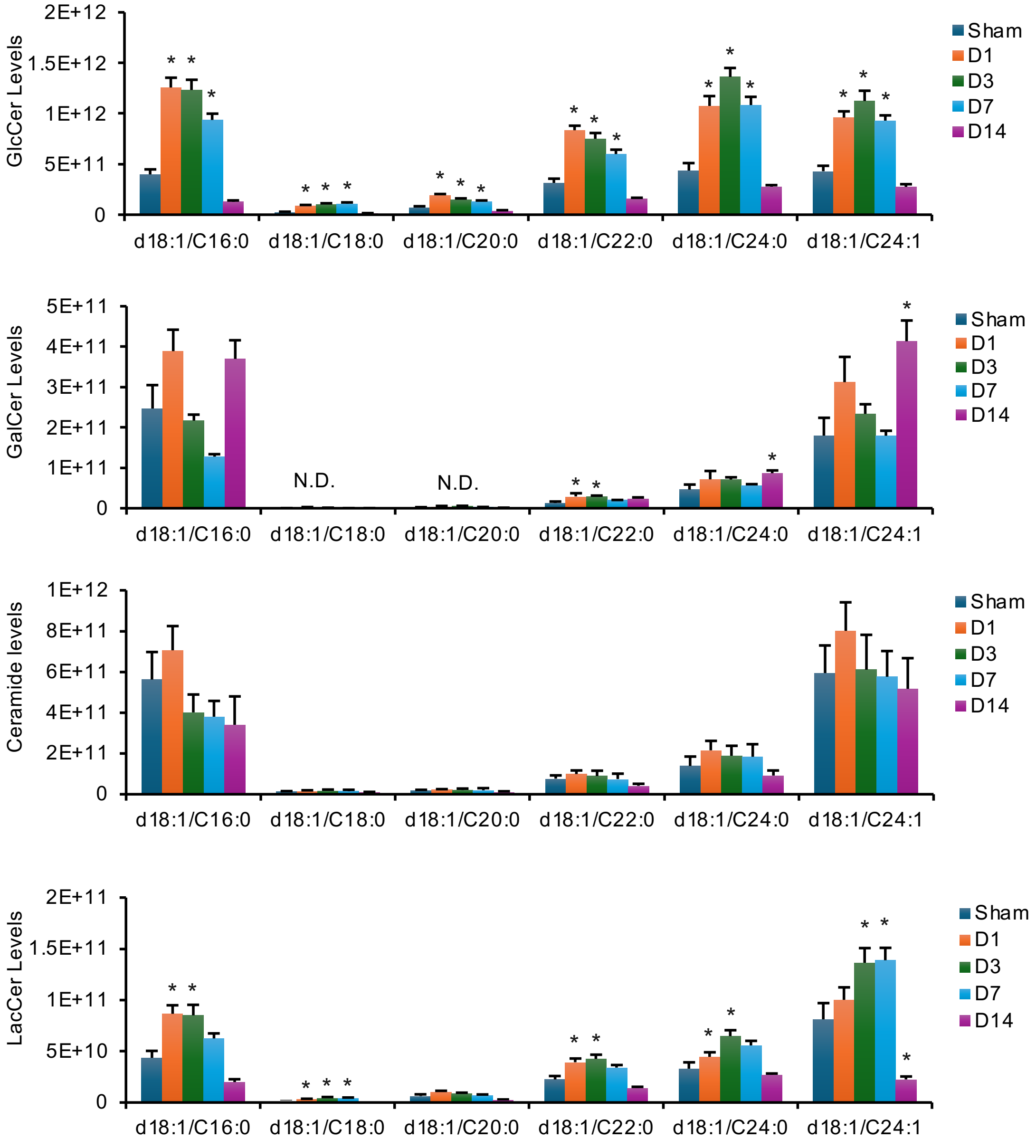

Supplementary Figure 1. Glycolipids levels after renal ischemia–reperfusion injury.

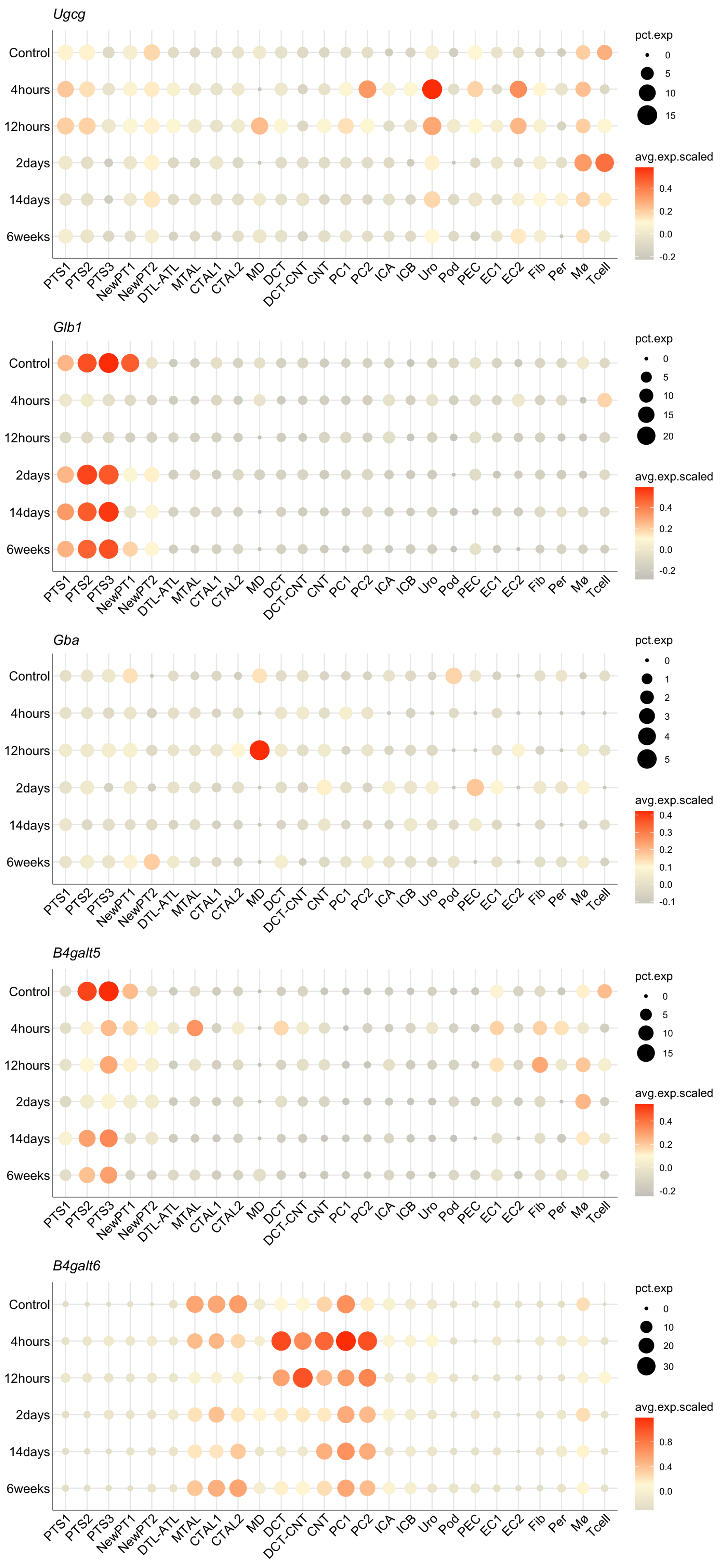

Supplementary Figure 2. Public snRNA data on expression levels of GlcCer metabolism–related genes during renal ischemia–reperfusion injury

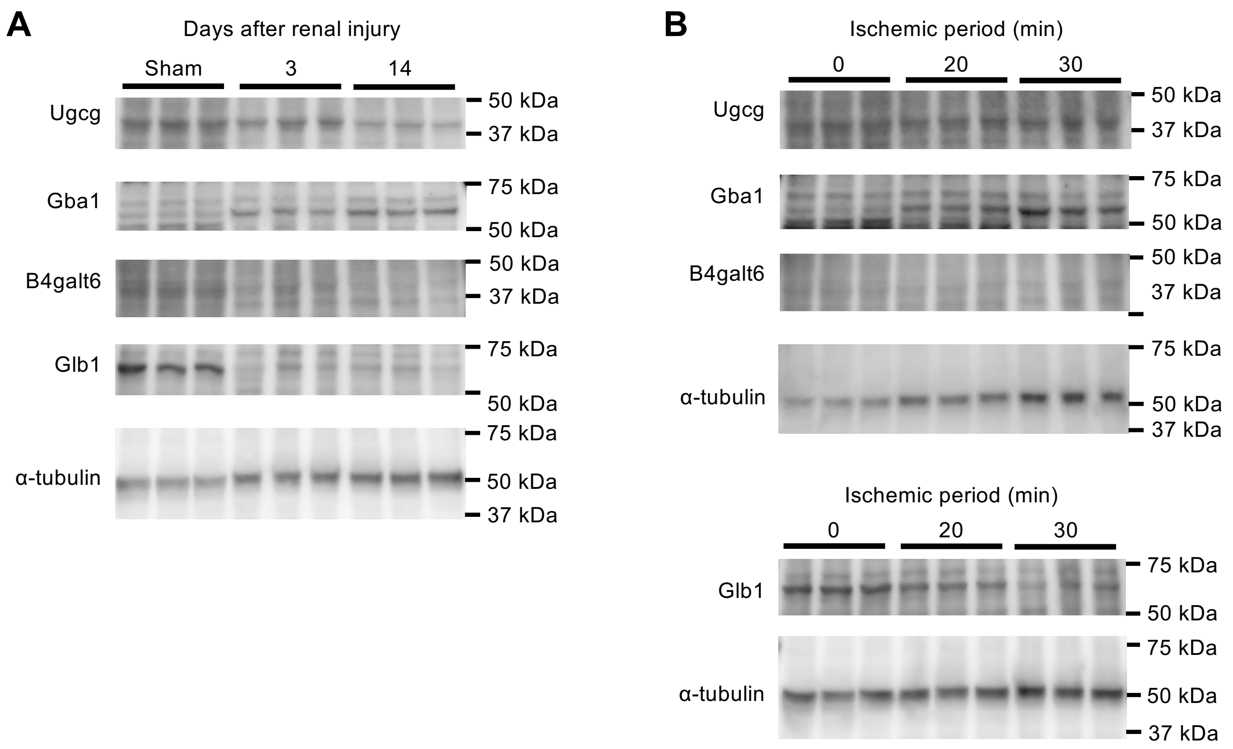

Supplementary Figure 3. Protein levels of GlcCer metabolism–related enzymes in the kidneys after ischemia–reperfusion injury

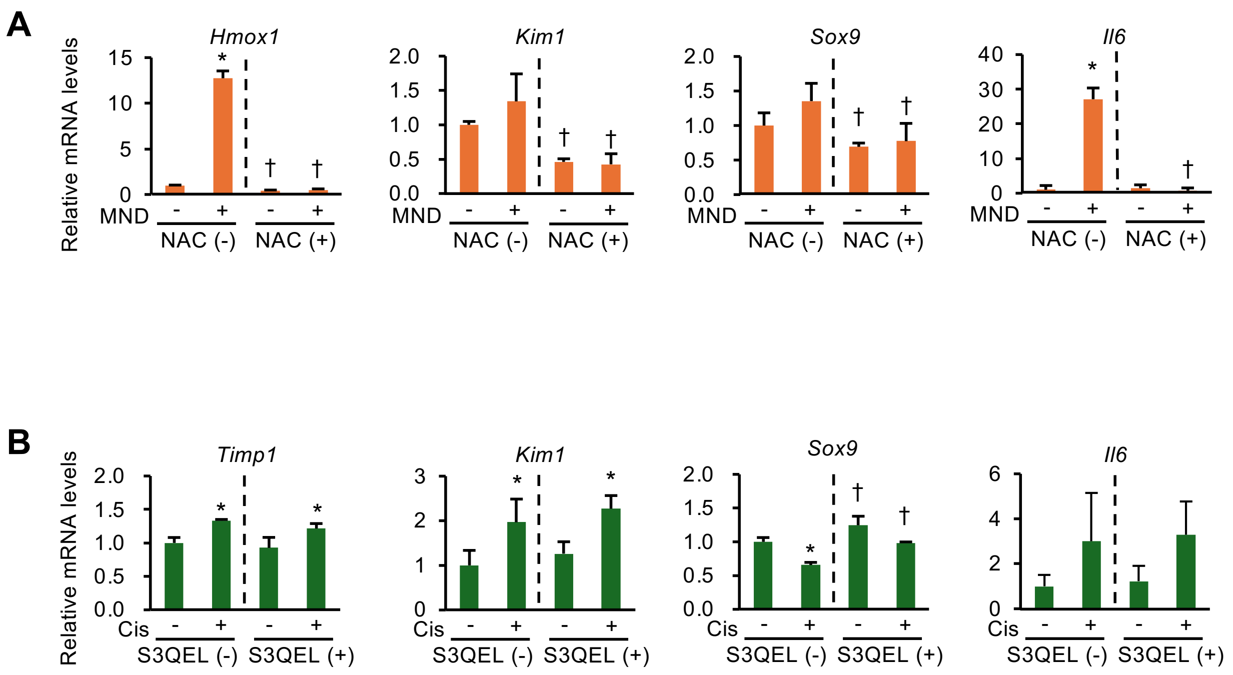

Supplementary Figure 4. Expression of genes in cultured renal tubular epithelial cells

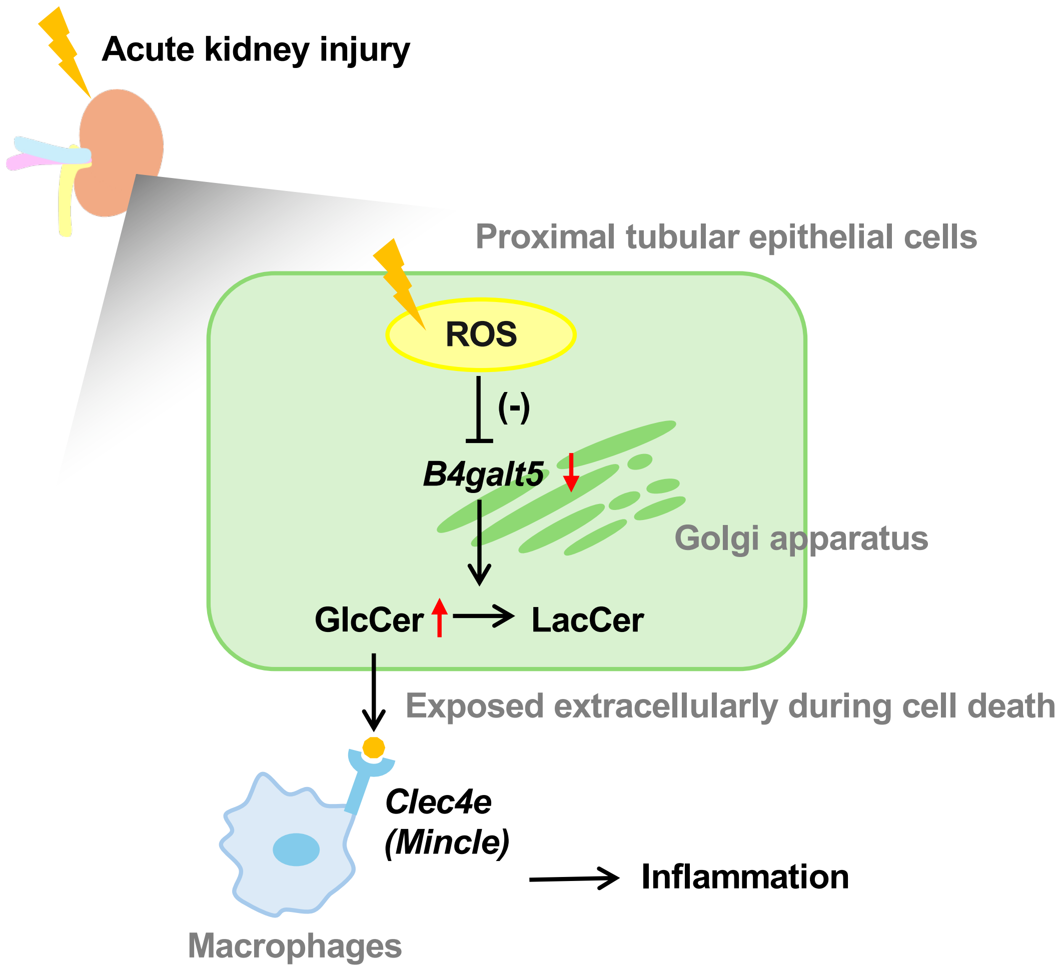

Supplementary Figure 5. Schematic illustration of this study

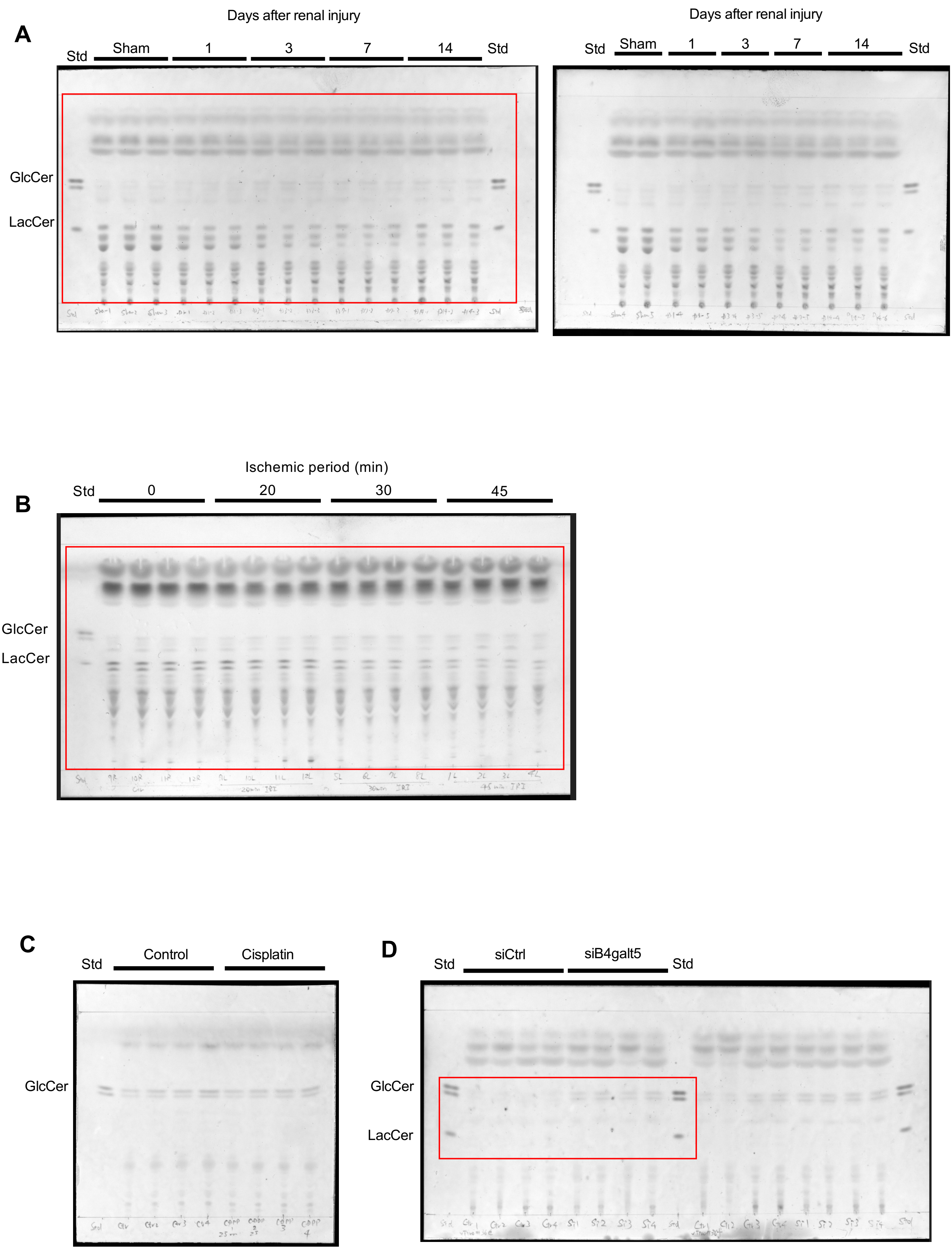

Supplementary Figure 6. Full images of TLC used for quantification in Figures 1A, F, 4B, and 5B, C.

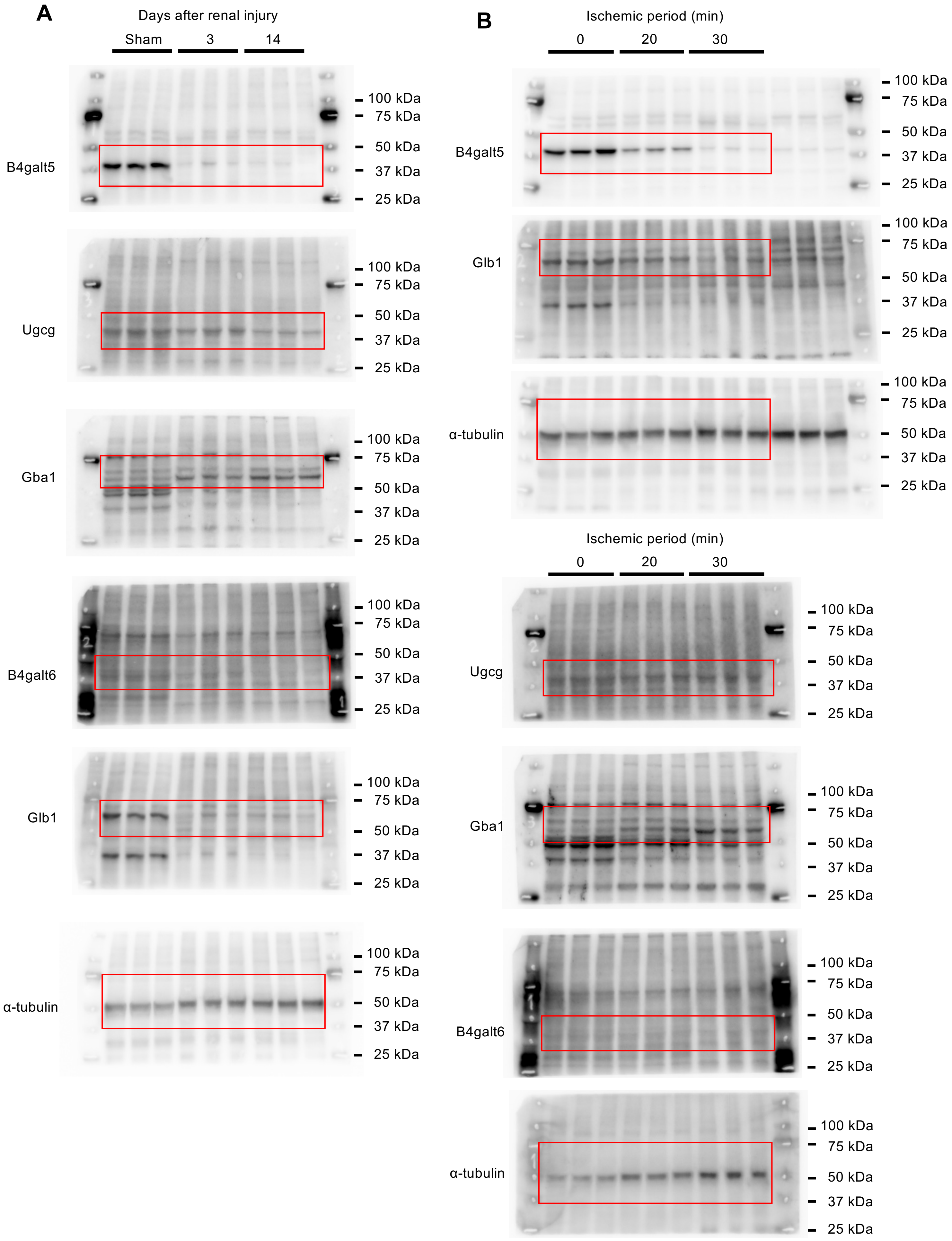

Supplementary Figure 7. Full images of Western blots used for quantification in Figure 2D, E and Supplementary Figure 3A, B.
